# Considerations in the assessment of heart rate variability in biobehavioral research

**DOI:** 10.3389/fpsyg.2014.00805

**Published:** 2014-07-22

**Authors:** Daniel S. Quintana, James A. J. Heathers

**Affiliations:** ^1^NORMENT, K.G. Jebsen Centre for Psychosis Research, Institute of Clinical Medicine, University of OsloOslo, Norway; ^2^Division of Mental Health and Addiction, Oslo University HospitalOslo, Norway; ^3^School of Psychology, University of SydneySydney, NSW, Australia

**Keywords:** heart rate variability, autonomic nervous system, parasympathetic nervous system, psychophysiology, respiration, emotion

## Abstract

Heart rate variability (HRV) refers to various methods of assessing the beat-to-beat variation in the heart over time, in order to draw inference on the outflow of the autonomic nervous system. Easy access to measuring HRV has led to a plethora of studies within emotion science and psychology assessing autonomic regulation, but significant caveats exist due to the complicated nature of HRV. Firstly, both breathing and blood pressure regulation have their own relationship to social, emotional, and cognitive experiments – if this is the case are we observing heart rate (HR) changes as a consequence of breathing changes? Secondly, experiments often have poor internal and external controls. In this review we highlight the interrelationships between HR and respiration, as well as presenting recommendations for researchers to use when collecting data for HRV assessment. Namely, we highlight the superior utility of within-subjects designs along with the importance of establishing an appropriate baseline and monitoring respiration.

## THE USE OF HRV IN EMOTION SCIENCE AND PSYCHOLOGY

The autonomic nervous system has been studied as a correlate of emotion for almost a century (Cannon, 1916). A central technique within this tradition of research is heart rate variability (HRV), which refers to a variety of methods for assessing the beat-to-beat change in the heart over time; these are used to approximate various aspects of autonomic outflow to the heart. Improvements in computing technology and miniaturization have made the electrocardiographic collection of inter-beat intervals (IBIs) accessible, and the analysis of the resulting beat-to-beat intervals trivial. One consequence of this access is a sustained interest in the application of HRV within the behavioral sciences, and in the psychology of emotion in particular. There are major biobehavioral theories that suggest that HRV can be used to investigate the central relationship between autonomic regulation and interpersonal interaction (Porges, 1995; Thayer and Lane, 2000). The neurovisceral integration model suggests that HRV is an index of the capacity for the central autonomic network (Benarroch, 1993) – which includes the brainstem, hypothalamus, and prefrontal cortex – to adjust to environmental demands (Thayer and Lane, 2000). Porges’ polyvagal theory takes a phylogenetic approach (i.e., it observes evolutionary and developmental commonalities within the structure and function of the vertebrate autonomic nervous system), arguing that social engagement is centrally facilitated by outflow and functional organization of vagus nerve (Porges, 1995). Consistent with this theory, reduced HRV has been observed in psychiatric disorders characterized by poor social cognition and emotion regulation (Bär et al., 2007; Quintana et al., 2013b). Interestingly, psychiatric patients also demonstrate less HRV reactivity during different levels of mental loading in comparison to healthy controls (Valkonen-Korhonen et al., 2003), further highlighting the poor cardiorespiratory regulatory capacity of this population.

While it may be the case that HRV can be used as a neurobiological index of interpersonal interaction, significant caveats exist due to the complicated nature of HRV and consequently uncertainty regarding what information is actually provided by common HRV indices (Berntson et al., 1997; Malpas, 2002; Billman, 2011). Additionally, the relationship between HRV and vagal modulation is complex in itself with a large interindividual variation (Picard et al., 2009). The problem is further compounded by the co-modulation of various respiratory and circulatory factors, which occur via numerous mechanisms and over multiple time-scales. Moreover, both breathing and blood pressure regulation have their own directly mediated relationships to the tasks employed in social, emotional, and cognitive experiments – if this is the case, we often have a complicated question of interlocking causalities. For instance, are observed changes in heart period epiphenomena that can be more parsimoniously described by changes in breathing or blood pressure? If the direction of causality between experimental task and the coordinated response within cardiac, circulatory, and respiratory variables is poorly understood, simple relationships between task and output changes may be obscured. Finally, experiments are often poorly designed as uncontrolled variables within typical experimental environments may drastically influence HRV. Few papers ideally control for medication, food, and water consumption, bladder filling, time of day, and other extraneous factors (Tak et al., 2009; Heathers, 2014). The overall aim of this review is to highlight the interrelationships between the nature and extraneous control of HRV, with a particular emphasis on respiration, and discuss implications for research in emotion science and psychology. Firstly, a number of important factors for the assessment of HRV in general and in emotion psychology in particular will be outlined. Secondly, solutions will be presented to reduce the potential impact of these factors.

## CAVEATS AND CONSIDERATIONS

### RESPIRATION IN HRV RESEARCH

Coupling between respiration and heart rate (HR) has a long research history, and was noted in classical animal studies pre-dating the electrocardiogram, which noticed fluctuations with breathing of heart beat and blood pressure (Ludwig, 1847). Consequently, the typically functioning respiratory system is presently characterized by complex breath-to-breath variations in respiratory rate and depth (Bruce, 1996) coupled with both heart period and blood pressure oscillations in a network of continual co-modification. For instance, a decrease in respiratory frequency generally corresponds with a lengthening of the heart period (Bruce, 1996). The traditional experimental approach of assessing the impact of the manipulation of one of these variables on another has led to important advancements in the understanding of cardiorespiratory coupling. However, perturbing the cardiorespiratory system does not allow the observation of casual relationships during spontaneous activity. Procedures developed to examine the coupling between time series may facilitate the identification of directionality and strength of cardiorespiratory coupling during spontaneous activity but these traditionally have only provided a limited insight into causality (e.g., Granger causality; Granger, 1969). Indeed, cardiorespiratory interaction has been variously quantified as primarily respiration-to-heart rate (Rosenblum et al., 2002; Zhu et al., 2013) heart rate-to-respiration (Larsen et al., 1999; Tzeng et al., 2003) or neither (i.e., bidirectional; Porta et al., 2013). These differences are likely to strongly depend on the analytical technique employed, but the details of this are unclear.

The nature of cardiorespiratory coupling is of intense research interest, highlighted most centrally by a robust debate concerning the central (Eckberg, 2009) and baroreflex (Karemaker, 2009) mechanism contributions to respiratory sinus arrhythmia (RSA). There is also a common genetic influence on HRV and respiration (Kupper et al., 2005). To further complicate this already complex relationship, the degree of cardiorespiratory coupling depends on the respiratory rate. That is, as the respiratory rate increases, HR increases phase distance from respiration. For instance, a breathing rate of 5–6 breaths per minute corresponds with a phase angle increase of 90°, continuing to a phase angle of 180° with 10 breaths per minute (Angelone and Coulter, 1964). Indeed, a presumed tenet of RSA – that shorter R-R intervals should be coupled with the apogee of inspiration – only occurs at a slow respiratory rate of six breaths per minute (Vaschillo et al., 2004), around half the natural respiration rate. However, there is no relationship between cardiorespiratory coupling and baroreflex sensitivity or blood pressure variability (Tzeng et al., 2003).

Further, shared neural networks for respiratory and HR oscillations (Evans et al., 2009) suggest that the manipulation on breathing may also lead to unintended effects on HRV by removing some of the variance in HRV that may relevantly covary with experimental task. Intriguingly, the degree of coupling may be higher when HRV is increased and at lower breathing frequencies (Galletly and Larsen, 2001; Tzeng et al., 2003), suggesting that unhealthy populations or experiments that are designed to reduce HRV may be more prone to decoupling of cardiorespiratory oscillations. This observation is particularly relevant when comparing two populations that may display different breathing frequencies (e.g., anxious vs. non-anxious participants) or when an experimental manipulation modifies respiration. Notably, respiration is not a necessary condition to modify HR over time as variability is still observed (although significantly reduced) without mechanical respiratory input to the heart (Larsen et al., 1999). Conversely, individuals with no vagal input to the heart (e.g., heart transplant recipients) still demonstrate RSA (although to a much smaller degree) presumably due to mechanical effects on the sinoatrial node (Bernardi et al., 1989; Slovut et al., 1998). While respiration influences blood pressure via mechanical intrathoracic pressure changes, this is buffered by HRV (Toska and Eriksen, 1993; Elstad et al., 2001). The influence of respiration on blood pressure is likely to be caused by the mechanical influence on venous return, modulating cardiac output (Triedman and Saul, 1994) via changes in stroke volume, which in turn influences blood pressure (Elstad et al., 2001).

### THE IMPACT OF RESPIRATION DURING SOCIAL-EMOTIONAL TASKS

Social-emotional tasks have been shown to reduce breathing variability (Vlemincx et al., 2011, 2012a), even for positively valenced emotions (Boiten, 1998), due to the “locked-in” attention often required during social-emotional tasks. Moreover, the mental stress that usually accompanies these tasks can also disorder general respiratory coordination (Vlemincx et al., 2012b). In addition to overall breathing variability, experimental stress induction can also influence the specific length of inspiration and expiration (Cohen et al., 1975). Thus, a social-emotional task that induces a change in respiratory time variables and/or depth may be indirectly influencing HRV. The rates of sighing also increase during these tasks (Vlemincx et al., 2011), with sighs shown to “reset” both respiratory variability and emotional states (Vlemincx et al., 2013). This is consistent with observations of increased sighing in a range of anxiety disorders (Abelson et al., 2001; Nardi et al., 2009), and increased sighing during experimentally induced stress (Vlemincx et al., 2012b). Finally, continual focused attention (e.g., during psychometrics tasks) has been shown in a number of studies (Mulder and Mulder, 1981; Aasman et al., 1987; Middleton et al., 1999) to reduce LF HRV, which creates further difficulties for interpretation.

There has been considerable debate on the necessity of controlling for respiration when assessing HRV. Denver et al. (2007) have argued against the need to control for respiration – at least for resting state recordings – given the important influence of breathing on HRV. To wit, by controlling for breathing in HRV recordings the researcher is removing an important influence on HRV (but see Grossman and Taylor, 2007). Denver et al. (2007) argue that if we assume that both respiration and heart beat oscillations are generated from the same central origin (e.g., Eckberg, 2009) then under resting state conditions controlling for respiration may not be necessary. Indeed, proponents for the control of respiration assume (either explicitly or implicitly) that alterations in respiratory frequency bring provoke HRV changes (i.e., the direction of causality moves from respiration to HR) without considering that HR adjustments may provoke changes in respiratory drive (Tzeng et al., 2003).

One compromise solution is to measure a participant’s natural breathing rate, and use the derived frequency for respiratory pacing (e.g., Elstad, 2012). While this approach has utility during resting state registration, this procedure may inadvertently influence HRV during emotional or cognitive tasks as the participant has to consciously follow the pacing cue, in addition to paying attention to the experimental task – dual attention, in a number of contexts, significantly increases task difficulty (Pashler, 1994). Zhang et al. (2010) argue that cardiorespiratory coupling during a cognitive task can be influenced either by activation of the motor cortex, which deceases cardiorespiratory coupling, or via increases in SNS activity from completing a cognitive task. However, here sympathetic outflow was indexed by normalized low frequency HRV – which is not straightforwardly related to SNS activity (e.g., Grassi and Esler, 1999; Moak et al., 2007; Goedhart et al., 2008; Billman, 2011, 2013a) – so this latter claim requires further empirical support using indices that more directly index cardiac sympathetic outflow.

Finally, slow respiratory rates (below 0.15 Hz) hinder the reliable estimation of RSA given the overlap with the LF component, which can be an issue for physically fit individuals, or with experimentally induced relaxation. While the RSA peak can be visually identified on a person-to-person basis, an objective algorithm based on a continuous wavelet transform has been developed to select variable HF bandwidth based on the power spectrum of the respiratory signal (Goren et al., 2006).

### THE POORLY ADDRESSED NATURE OF HRV

While the collection of raw interbeat interval data is relatively straightforward process, several lines of evidence suggest that ancillary and interpretative factors surrounding HRV receive insufficient attention.

(1) Heart rate variability is affected by respiratory depth (Hirsch and Bishop, 1981) and frequency (Angelone and Coulter, 1964; Brown et al., 1993). Specifically, greater RSA magnitude occurs during higher tidal volumes and lower respiratory frequencies. In addition, basal respiratory frequency has a non-linear relationship with spectral power as breathing rate falls below approximately 0.15 Hz (as it occasionally does in athletes; Saboul et al., 2014). Thus, any task that increases respiratory tidal volume and/or reduces respiratory frequency (e.g., meditation; Krygier et al., 2013), or conversely decreases tidal volume and/or increases respiratory frequency (e.g., mental stress; Houtveen et al., 2002) is likely to indirectly modify HRV. More recently, it has also been shown that the inspiration:expiration (I:E) ratio also effects HRV (Strauss-Blasche et al., 2000). Specifically, HRV increases when short inspiration is followed by long expiration – which has implications for tasks that require speech production (Cysarz et al., 2004) and many forms of meditation, for instance. Even monitoring spontaneous breathing has been found to reduce respiratory variability (Cysarz and Büssing, 2005; Conrad et al., 2007). HR driven cardiorespiratory coupling also appears to increase when HRV is higher (Galletly and Larsen, 2001).

(2) While respiration has been most typically studied as the dominant physiological rhythm relevant to HRV, much less in known about chemosensory (Berthoud and Neuhuber, 2000; Niewinski et al., 2014) and circadian (Furlan et al., 1990; Guo and Stein, 2002; Bonnemeier et al., 2003) influences.

(3) Heart rate variability continues to be used to form an index of putative autonomic outflow by measuring a point on a simple continuum of parasympathetic/sympathetic activity. While this model is still popular, it is directly at odds with a great deal of available evidence; for instance, that neuropeptide Y directly mediates transmission between adrenergic and muscarinic neurons (Revington and McCloskey, 1990). This approach, generally focused around the use of the LF/HF ratio (the ratio of low frequency power to high frequency power) to represent “sympathovagal balance,” has been criticized extensively for over two decades (e.g., Eckberg, 1997; Billman, 2013a). This obscures the interpretation of HRV from the approximately 65% of papers which still report metrics in this manner (Heathers, 2014). While it is clear that LF power does not represent sympathetic activity (Goldstein et al., 2011) it is important to note that there has also been robust debate surrounding the relationship between HF power and parasympathetic activity (for a review see Billman, 2011).

(4) Differences in the prevailing HR can influence HRV both mathematically, due to the inverse curvilinear relationship between HR and RR interval (Sacha and Pluta, 2008) and physiologically, via the augmenting or diminishing effect of the autonomic constituent of HRV (Billman, 2013b). Consequently, emotional interventions that reduce PNS activation could inflate reductions in HRV via HR increases that are independent of changes in cardiac autonomic nerve activity. Nevertheless, it is possible to mathematically correct for the influence of the prevailing HR on HRV (Sacha, 2013; Pradhapan et al., 2014), which may also improve the reproducibility of HRV (Sacha et al., 2013).

Notwithstanding the evidence, these important caveats do not discourage research in the social and psychological sciences, which equate HRV variously as an index of emotional regulation (Appelhans and Luecken, 2006), stress response (Berntson and Cacioppo, 2004), and interpersonal engagement (Butler et al., 2006). Moreover, over 32 studies have specifically investigated the effect of emotion on HRV in healthy participants (Kreibig, 2010).

### THE NON-LINEAR NATURE OF HRV

Frequency analysis assumes the HR signal is stationary (Stratonovich, 1967) and that over time it can be modeled as the sum of cyclical processes, but this is demonstrably not the case. While removing slow or DC trends from short periods of HRV will create a quasi-stationary series (e.g., Tarvainen et al., 2002), HRV in general displays the characteristics of a non-linear signal, given the biological origin and the origin of HRV deriving from sum of processes that operate on a variety of time scales (Winfree, 2001; Piskorski and Guzik, 2007; Stein et al., 2008). The non-linear interaction of the PNS and SNS systems may also contribute to heart beat complexity observed in healthy participants (Levy, 1971). 1/*f*-like scaling of the heart beat signal, which is characteristic of a heart beat series from a healthy individual (Ivanov et al., 1999; Goldberger et al., 2002), also points to a non-linear basis. A 1/*f* scaling of the heart beat signal (α = 1) falls exactly between a completely random signal (α = 0.5; i.e., white noise) and an entirely predictable signal (α = 1.5). For instance, pathological heart rhythms tend to demonstrate Brownian noise (Peng et al., 1995). A complex interaction of linear and non-linear systems contribute to HRV (Voss et al., 2009), which suggests that measures of complexity may be a better measure of autonomic nervous system outflow (Kaplan et al., 1991). Indeed, non-linear measures of HRV have demonstrated improved prognostic information in heart failure patients with in comparison to linear HRV measures (Bigger et al., 1996; Huikuri et al., 2000). However, the utility of non-linear HRV measures have been questioned due to a lack of reproducibility (Tan et al., 2009).

Intriguingly, non-linear analysis indicates that some elderly patients with cardiovascular disease unexpectedly display increased HRV indices (Stein et al., 2005) due to erratic, non-respiratory sinus arrhythmia. These erratic rhythms have also been found to predict the onset of ventricular tachycardia (Mäkikallio et al., 1997) and mortality post-myocardial infarction (Stein et al., 2008). The source of this erratic non-respiratory sinus arrhythmia may be due to increased sympathetic activity (Tulppo et al., 1998), which is consistent with the higher concentrations of plasma noradrenaline observed in patients post-myocardial infarction (Christensen and Videbaek, 1974). Alternatively, erratic rhythms may be caused by poor coordination between the sinoatrial and atrioventricular nodes, which could reflect a pre-clinical manifestation of sick sinus syndrome (Stein et al., 2008).

**FIGURE 1:**
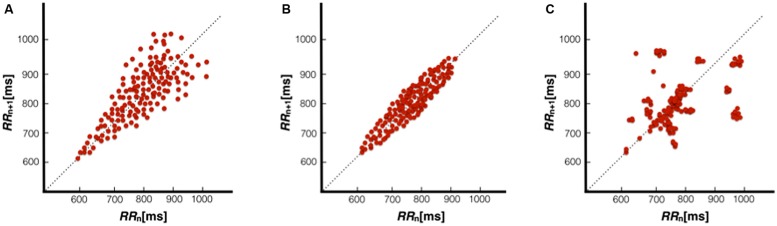
**Poincaré plots representative of a healthy individual with a comet pattern (A), heart failure patient with a torpedo pattern (B), and heart failure patient with a complex pattern (C).** Figures adapted from Woo et al. (1992).

A Poincaré plot is a visual, non-linear HRV index comprised of points that represent two consecutive heart periods, with any point above the identity line (a 45° slope that passes through the origin, which represents equal consecutive heart periods) representing a longer heart period, whereas points below the identity line represent a shortening of the heart period. A healthy participant typically displays a “comet” shaped plot (**Figure 1A**), with a wider dispersion of points as the beats lengthen. Even at different rates of breathing (ranging from 6 to 16 breaths/min) this shape persists in healthy participants (Guzik et al., 2007). On the other hand, patients with heart failure display atypical “torpedo,” “fan,” or “complex” (i.e., stepwise clusters of points) patterns (Woo et al., 1992). A torpedo shape (**Figure 1B**) is indicative of a lack of R-R interval increase when HR slows, whereas fan and complex patterns (**Figure 1C**) may represent general issues with cardiac autonomic regulation. Poincaré plots have been demonstrated to shown to display significant asymmetry in approximately 80% of individuals (Guzik et al., 2006; Piskorski and Guzik, 2007; Porta et al., 2008), with the plot “cloud” above the identity line appearing larger than the plot cloud below the line. Absent of long-term trends or very low frequency (VLF) power changes typically removed via detrending or high-pass filtering, HR acceleration will be matched with a roughly corresponding deceleration over time, and the Poincaré plots might be expected to be symmetrical. However, this commonly observed asymmetry in Poincaré plots suggests that HR accelerations operate in a different manner than decelerations, possibly due to baroreflex responses (Guzik et al., 2006). While the source of this asymmetry is unclear, it reinforces the fact that HRV is generated by complex non-linear dynamics. Together, this work emphasizes the importance of scrutinizing Poincaré plots for irregularities, particularly for populations characterized by low HRV (e.g., older participants), and urges caution with the central assumption that IBIs over time can be meaningfully devolved into the sum of sine waves as in traditional frequency-domain analysis.

### EXTERNAL FACTORS THAT CAN INFLUENCE HRV

A number of external factors are usually controlled for in HRV research, including the intake of nicotine (Hayano et al., 1990; Sjoberg and Saint, 2011) and caffeine (Sondermeijer et al., 2002) preceding data collection. Cardioactive medication use, including some antidepressant classes (e.g., tricyclics; Kemp et al., 2010), some antipsychotic classes (e.g., clozapine; Cohen et al., 2001), benzodiazepines (Agelink et al., 2002), and antihypertensives (Schroeder et al., 2003) are also usually accounted for, although this may be somewhat difficult in practice when testing patient populations. Other factors that are usually accounted for include the time of day (Massin et al., 2000; van Eekelen et al., 2004), levels of habitual alcohol use (Quintana et al., 2013a,b), physical activity levels (Britton et al., 2007; Soares-Miranda et al., 2014), and age (O’Brien et al., 1986). Digestion of food and water are less commonly accounted for in HRV research, but both provoke a coordinated autonomic response. For instance, digesting food has been shown to reduce parasympathetic activity, even an hour after eating a 500 kcal meal (Lu et al., 1999). Even exposure to food-related cues elicits a similar response (Nederkoorn et al., 2000), suggesting a physiological response to the anticipation of a meal. Conversely, missing a meal (i.e., fasting) appears to have its own coordinated effects on HRV (Pivik et al., 2006), supporting the recommendation that participants consume a light meal approximately 2 h before the assessment of HRV (Tak et al., 2009). Water consumption has also been shown to increase HF-HRV in particular (Routledge et al., 2002), due to the vagal buffering response to the pressor effect provoked by hypo-osmotic fluids (Scott et al., 2001). Notably, this buffering response to the pressor effect is attenuated in older individuals (Jordan et al., 2000) and not observed in those with cardiac vagal denervation (Routledge et al., 2002). In addition, both bladder and gastric distension can also have an appreciable influence on HRV; these have been associated with increases in blood pressure and sympathetic outflow (Fagius and Karhuvaara, 1989; Rossi et al., 1998). However, papers only very rarely report that participants were asked to empty their bladder before experimental participation (Heathers, 2014).

## POTENTIAL METHODOLOGICAL CONTROLS

### WITHIN-SUBJECTS DESIGN OFFERS OPTIMAL EXPERIMENTAL CONTROL

In light of the complex interactions described above, a within-subjects design is the most appropriate method to explore the role of cardiorespiratory oscillations on behavior. Indeed, to appropriately detect a difference between groups, a sample size between 30 and 77, depending on the HRV metric used, is needed (Pinna et al., 2007). However, subgroups are commonly employed in these designs (e.g., gender, psychiatric comorbidities), which have been suggested to require 20 participants per cell (Simmons et al., 2011). Although some contexts make this difficult (i.e., comparison of psychiatric groups), within-subjects is the ideal design. The use of within-subjects design can eliminate any interindividual differences in coupling between HR, BP, and respiration. For instance, approximately 30% of individuals do not demonstrate any discernable synchrony between respiration and HR (Schäfer et al., 1998; Tzeng et al., 2003), with cardiorespiratory synchronization less likely to occur during higher breathing frequencies. While it is debatable if respiration should be controlled in HRV recordings, it is clear that sighs and long breaths have an effect on HRV as they generate non-sinus rhythm HR.

All of the caveats above can be minimized when individual comparisons are made between experimental points, that are as similar as possible. Most importantly, in the context of HRV, within-subjects designs better facilitate; (i) the removal of participants with atrial premature complexes and ventricular premature contractions, along with sighs, coughs, and gasps as such phenomena are easier to identify from multiple recordings if these are regular electrocardiographic errors or habitual behaviors; (ii) the elimination of individual differences in respiration rate, along with the avoidance of potential non-linear relationship of individual differences in respiration/HR relationship; (iii) the need for less participants (and consequently improved control over external variables due to repeat attendance under identical conditions); and (iv) a reduction in the impact of external factors such as medication, alcohol, nicotine, and recreational drug use.

### DEFINING A “RESTING STATE” OR BASELINE

In an attempt to measure the effect of psychological task or group designation, much research assesses HRV during a resting state as a comparison to intervention. While informative, a more suitable method to interpret complex relationships between autonomic phenomena and psychological processes may be to perturb the cardiac autonomic system from complete rest. However, what constitutes a baseline needs to be carefully addressed depending on circumstances. A within-subjects experiment offers the most amount of control as a baseline is more likely to be similar.

Several caveats exist to the establishment of a baseline as an appropriate point of comparison. Firstly, the baseline HR needs to be able to support the respiratory signal without aliasing (Witte et al., 1988) – for instance, a normative breathing rate of 0.3 Hz can only be observed successfully in a HR faster than 0.6 Hz (i.e., 36 bpm). In a regular ECG, this criterion is often met. However, during supine recording, transient beats and intervals in healthy young people are frequently below 0.8 Hz (i.e., 48 bpm) – this may extend up to the entire IBI series in the case of physically fit individuals or any other participant displaying bradycardia. This corresponds with the fastest criterion for RSA in the HF-HRV band (i.e., 0.4 Hz). While this is an abnormal situation (see Sacha and Grzeszczak, 2002), it is a potential confound to the establishment of a baseline, especially if IBI series are filtered incorrectly (Grossman and Taylor, 2007). Secondly, physically fit participants may not have sinus rhythm appropriate for analysis in the first instance due to potential changes to the sinoatrial node – hearts of such individuals have often been assumed to be slower at rest due to higher vagal tone but the balance of evidence does not presently favor this explanation (Boyett et al., 2013). However, the resumption of “normal” sinus rhythm may be observed during exercise, orthostatic stress, etc. – if this is an experimental condition, then the transition from resting baseline is affected. Thirdly, tasks often compare passive eyes-open rest as a baseline to the performance of a psychomotor, attentional, or emotional task, for instance. It is possible that this conflates the difference between passive rest vs. the act of paying attention to task with the difference between passive rest vs. the specific task demands of the experiment in question. A popular alternative to complete rest is the Vanilla baseline (Jennings et al., 1992), which requires subjects to perform a trivial counting task requiring sustained attention but minimal cognitive load, as opposed to what the authors term “enforced relaxation.” Other similar approaches have been attempted (e.g., Piferi et al., 2000). Finally, with individual recordings made over time, there is the complicated situation of the immediacy of baseline-to-experiment transition. HR is not stable over time, and can exhibit non-periodic phenomena or bifurcations, which may be in conflict with the assumption that an initial baseline well reflects a later experimental condition. Researchers must also consider the potential effect of decay between tasks if cardiorespiratory effects are observed, what a normalization to baseline might look like, and of course the fact that secondary baselines may conflict with experimental instructions or manipulations. It is inherent from the above that an appropriate baseline is not a singular measurement with “correct” parameters under all circumstances, but rather the non-task situation that best controls for the presence of task comparison. In many situations, the comparison of a task to a “resting” state will therefore vary in appropriateness.

### MONITORING RESPIRATION

As detailed above, basic changes in respiration can have a significant impact on HRV. Pneumotachography is the gold standard for the monitoring of tidal volume, however, the use of a closed face-mask required to do so is cumbersome and impractical for most research in emotion and psychological science (e.g., face-to-face interactions). In lieu of this, the use of a strain gage to index the expansion of the chest can give sufficient information – most importantly, a strain gage can identify gross deviations of typical cyclical respiration (e.g., sighs, coughs). Mirroring the importance of HR measures to reflect true sinus rhythm (as an ectopic beat does not represent ANS input to the SA node), “true” respiratory cycles must also be used to correctly draw inference on respiratory oscillations and coupling to HR. However, signals from strain gages do not necessarily have a linear relationship of circumference to signal (i.e., distension/signal output relationships may be different at different belt tensions) and that chest circumference is itself an indirect measure of the respiratory cycle (i.e., lung and chest wall volumes are not identical). In lieu of direct respiratory measures, established algorithms (Moody et al., 1985, 1986) that have been successively improved (e.g., Park et al., 2008; Langley et al., 2010) can also provide an appropriate surrogate measure of respiration from based on ECG signal morphology.

There is at present no satisfying solution for a totally non-invasive monitoring of tidal volume but in the meantime it seems prudent to monitor respiration at least to identify gross errors from normal cardiorespiratory analysis assumptions. For instance, healthy participants occasionally breathe at frequencies slower than 0.15 Hz (up to 35% of participants; Hoit and Lohmeier, 2000; Beda et al., 2007; Pinna et al., 2007) – this has also been observed in physically fit individuals (Saboul et al., 2014). Breathing below 0.15 Hz dramatically increases the observed power of RSA over that of typical breathing frequencies due to the involvement of the baroreflex. Consequently, this will dramatically affect measures of LF-HRV, HF-HRV, total spectral power and any ratio between spectral bands (e.g., LF/HF or LFnu). However, any such participant can be easily identified from the unitless cyclical information provided by a strain gage, and subsequently discarded from analysis. Alternatively, it may be possible to remove the immediate effect of slow breathing cycles using a continuous wavelet approach (or any spectral analysis method that handles discontinuity well, such as an averaged lomb-scargle periodogram) to identify affected areas. Naturally, areas affected by slower breathing can be compared within subjects to periods of regular sinus rhythm (if available) to determine the level of distortion present in either spectral band. At present, we are not aware of any work that proposes an acceptable amount of distortion.

## CONCLUSION

Enthusiasm for HRV within emotion science is subsequent to it being seen as a source of accurate, cheap, and non-invasive insight into autonomic outflow. This position should be strongly tempered by the present considerations. Instead, it would be more reasonable to say that HRV presents an admixture of insight and significant layers of complication. The behavior of the heart over time is the end-state of multiple interlocking systems, which present their own individual challenges for researchers at a cellular, local, and systemic level.

It should be mentioned here that while this paper focuses solely on issues of traditional methodological control, there are other domains in which significant improvements in the experimental environment surrounding HRV might be gained. Most crucially, signal analytic requirements often receive surprisingly little attention, and decisions about type of spectral analysis, windowing, and data cleaning are crucial (e.g., Berntson and Stowell, 1998) but are often under-reported. Likewise, recent interest in data uploading and retention (e.g., Nosek et al., 2012) has received little systematic attention in cardiac psychophysiology so far, even though a) data retention is a American Psychological Association requirement (American psychological association [APA], 2001) and b) the ability to broadly access raw data is a potentially excellent control for the methodological and analytical issues outlined here, as well as a test bed for the development of future HRV metrics and meta-analysis.

The best case scenario for the continuing use of HRV is that the significant challenges and complications provided by interrelationships most crucially between respiration and blood pressure are acknowledged, and that experimental designs are improved by appropriately accounting for common external factors known to aggressively modify HRV. Careful consideration of these factors will help ensure researchers use more accurate and reproducible measures of autonomic outflow.

## Conflict of Interest Statement

The authors declare that the research was conducted in the absence of any commercial or financial relationships that could be construed as a potential conflict of interest.
